# Shaping Workspaces, Shaping Lives: Health Implications of Working From Home for Employees With Tertiary Education in Switzerland

**DOI:** 10.3389/ijph.2026.1608002

**Published:** 2026-02-13

**Authors:** Szilvia Altwicker-Hámori, Sarah Heiniger, Marc Höglinger

**Affiliations:** Winterthur Institute of Health Economics, School of Management and Law, ZHAW Zurich University of Applied Sciences, Winterthur, Switzerland

**Keywords:** COVID-19 Social Monitor, fixed-effects estimation, occupational health, Switzerland, working from home

## Abstract

**Objectives:**

This study aimed to explore the effect of transitioning to working from home (WFH) on health for employees with a tertiary degree.

**Methods:**

Data were drawn from the COVID-19 Social Monitor, a large, high-frequency longitudinal online panel of the Swiss 18–79-year-old resident population (*N* = 3,381). We estimated individual-fixed-effects models to examine the effect of transitioning to WFH on 13 binary health outcomes related to general health, mental health, physical health, health behaviour and social trust.

**Results:**

Even post-COVID-19 WFH measures, the proportion of tertiary-educated employees working from home remained high relative to pre-pandemic levels. Individual fixed-effects estimates suggest no evidence of an effect of transitioning to WFH on any of the health outcomes.

**Conclusion:**

The upward trend in WFH underscores the importance of health-impact research in this context. The absence of adverse health effects is significant for employers and policymakers aiming to provide flexible work arrangements. Our study provides a benchmark for future research by encompassing a comprehensive range of health outcomes and utilizing a longitudinal panel structure that captures the transition from mandatory to optional WFH arrangements.

## Introduction

Working from home (WFH) has become a common option and even the “new normal” for a large proportion of employees in Switzerland [1]. Given that approximately 38% of Switzerland’s workforce is expected to work from home in 2030 [2], it is crucial to understand the impact of WFH on employee health. Therefore, this study aimed to explore how WFH affects the health of employees with a tertiary degree–the demographic group with the highest likelihood of WFH feasibility and adoption [1–3].

The richness of the dataset used in the analysis enabled us to adopt a holistic approach to defining and analysing health. This aligns with the definition of the World Health Organization (WHO): “Health is a state of complete physical, mental and social wellbeing not merely the absence of disease or infirmity” [4]. In particular, we examined 13 health outcomes, encompassing general health, mental health, physical health, health behaviour as well as social trust. While general health, mental health and physical health are commonly studied dimensions of health, the latter two have received less attention in the current context [5]. Addressing physical inactivity, a critical aspect of health behaviour, is particularly important given its substantial contribution to cardiovascular diseases [6], which rank as the fourth leading risk factor for mortality globally [7]. In Switzerland, cardiovascular diseases accounted for even the largest proportion of deaths at 27.5% in 2022, ahead of cancer [8]. Incorporating social trust is equally important in this framework, given the evidence that social trust contributes to increased levels of happiness and wellbeing [9]. Moreover, it is essential to consider social trust in light of research linking telecommuting to the quality of workplace relationships [10, 11], and the subsequent link between workplace relationships and overall health outcomes [12].

Considering a broad spectrum of outcomes is also important in view of the mixed international empirical evidence in this field, which varies depending on the specific health outcome studied [1, 13–19]. For example, a systematic review by Wilms et al. on the effect of the relocation of work to home during the COVID-19 pandemic found a decrease in physical activity, an increase in pain and a decrease in wellbeing [19]. Conversely, a Swiss study by Heiniger and Höglinger found a slight increase in physical activity and no significant effect of transition to WFH on neck pain or wellbeing [1]. Evidence regarding the impact of WFH on sleep during the COVID-19 pandemic also varies. For instance, a study by Staller et al. found that sleep efficiency, sleep duration, variability of sleep timing and social jetlag did not differ between those working from home and onsite in a German sample of students and employees [16]. In contrast, a Swedish study by Hallman et al. found that WFH was associated with longer duration of sleep than days working at the office, suggesting potential health benefits [17]. In terms of social relationships, a meta-analysis by Gajendran and Harrison found no generally detrimental effects of telecommuting on the quality of workplace relationships [10]. A qualitative study by Lal et al. in contrast highlight the difficulty in maintaining social interactions via technology, citing factors such as the absence of cues and emotional intelligence; nevertheless, some study participants expressed apprehension about returning to the traditional office environment, where social interactions may be viewed as distractions [20].

By focusing on the subpopulation with the highest likelihood of WFH feasibility and adoption and a comprehensive range of health outcomes, we aim to add nuanced yet comprehensive insights to the body of mixed evidence that extend beyond the COVID-19 pandemic period.

## Methods

### Data and Sample Selection

Data were drawn from the COVID-19 Social Monitor (Social Monitor henceforth), a large longitudinal online panel of the Swiss resident population aged 18 to 79 (*N* = 3,381), encompassing 24 survey waves from 30 March 2020 to 14 November 2022 [21]. The Social Monitor covered various public health issues, utilizing a questionnaire that mainly included validated items from established population surveys, such as the Swiss Health Survey (SHS) [22].

Participants of the Social Monitor were sampled from an existing online panel. The members of this panel were recruited using random probability sampling from national landline telephone directories and random digit dialing of mobile phone numbers. Participation in the Social Monitor was voluntary, and participants could always withdraw. The initial Social Monitor sample included 2,026 respondents in March 2020 and was supplemented with 1,355 additional respondents in December 2020. Surveys were conducted every two to 17 weeks. Additional study methodology, design details and baseline sample characteristics are available in the paper by Moser and co-authors [23]. The data can be accessed at https://doi.org/10.48620/358 [24].

In the current study, we included 651 participants who were employed and held a tertiary degree at the participants’ initial engagement in the Social Monitor survey. While restricting the analysis to tertiary graduates allows us to examine the subpopulation with the highest likelihood of WFH feasibility, it limits the generalizability of the results to the Swiss working population as a whole. Table 1 provides a description of the sample.

**TABLE 1:** Sample description (COVID-19 Social Monitor, Switzerland, 2020–2022).

​	Frequency	Percent
Age		
18–29 years	167	26%
30–39 years	180	28%
40–55 years	235	36%
56–65 years	66	10%
Missing values	3	0%
Sex		
Male	354	54%
Female	297	46%
Household income (CHF)		
<5,000	77	12%
5,000–9,999	262	40%
≥10,000	259	40%
Missing values	53	8%
Place of residence		
Urban	545	84%
Rural	106	16%
Working time		
Part time (≤49%)	48	7%
Part-time (50%–89%)	161	25%
Full-time (90%–100%)	422	65%
Missing values	20	3%
Survey entry		
Initial sample	382	59%
Additional sample	269	41%

Percentages are rounded to whole numbers.

### Variables

#### Independent Variable

Questions regarding WFH were developed specifically for the Social Monitor. For the descriptive analysis, we used the responses to two questions regarding WFH: (1) “Have you worked from home in the last 7 days?” and (2) “Did you work from home before the COVID-19 crisis?”. The response options “never”, “partially”, “mainly” and “exclusively” were considered separately in the descriptive analysis. In all fixed-effects models, we employed the transition to WFH as a binary predictor (never versus partially/mainly/exclusively) based on the former question.

#### Outcomes

We included 13 dichotomous outcome variables related to general health, mental health, physical health, health behaviour and social trust. All outcome variables, except two in the health behaviour domain specifically designed for the Social Monitor, are validated items derived from established population surveys. These surveys include the Swiss Health Survey [22], the Swiss Household Panel [25], the European Social Survey [26] and a validation study by Wanner et al. [27]. For all the outcome variables, the values were dichotomized so that a value of one indicates the presence of the worst (or worse) state. Detailed information about the variables, including the original survey question and response options as well as the source of existing items are presented in Supplementary Table S1. In the following, we present a summary of each dichotomous outcome variable categorized by the health domain.

First, to explore current general health, we employed two indicators: poor quality of life (very bad/bad versus neither good nor bad/good/very good) and poor self-rated health (very poor/poor versus average/good/excellent).

Second, to capture mental health in the past 7 days, we used two indicators: experiencing frequent stress (very often/frequently versus sometimes/rarely/never) and heightened strain (high versus middle/low). The latter variable is based on the Mental Health Inventory-5 (MHI-5) combining assessments of the following five items: (1) “very nervous”; (2) “so down or blue that nothing could cheer you up”; (3) “calm, balanced and serene”; (4) “discouraged and depressed” and (5) “happy” [28, 29]. Participants rated the negatively-worded items on a six-point scale, ranging from one (always) to six (never). The rating scale was reversed for the positively-worded items (i.e., “calm, balanced and serene” and “happy”), where one represented “never” and six represented “always”. To calculate the total MHI-5 score, the scores across all five items were summed up, the raw score was transformed to a 0–100 scale using the formula: (Actual raw score–lowest possible raw score)/Possible raw score range x 100. A score below 53 on the MHI-5 is considered highly clinically significant for mental disorders and was used as the threshold for dichotomization.

Third, to capture physical health in the last 7 days, we used five indicators: the presence of a headache (strongly/a little bit versus not at all), neck pain (strongly/a little bit versus not at all), back pain (strongly/a little bit versus not at all), sleep problems (strongly/a little bit versus not at all) and lack of energy (strongly/a little bit versus not at all).

Fourth, to integrate health behaviour, we utilized three indicators: complete physical inactivity in the last seven days (zero days of physical activity lasting at least 30 minutes versus one to seven days of physical activity lasting at least 30 minutes), frequent online-gambling in the past 14 days (multiple times a day/once a day/several times a week versus less than once a week/never) and frequent use of sleeping pills and sedatives in the past 14 days (daily/several times a week versus once a week/less often/never). It is important to note that the Social Monitor aimed to capture physical activity of at least moderate intensity by asking participants the following question: “On how many days were you physically active for a total of 30 min or more, causing you to breathe somewhat harder?” Examples of such activities included sports, exercise, training, as well as brisk walking or cycling, either for leisure or to get from one place to another.

Low social trust was included as our final variable based on the Social Trust Scale [30]. Participants were asked to respond to a battery of three questions about social trust: (1) “Would you say that most people can be trusted, or that you cannot be too careful in dealing with people?”, (2) “Do you think that most people would try to take advantage of you if they got the chance, or would they try to be fair?” and (3) “Would you say that most of the time people try to be helpful or that they are mostly looking out for themselves?”. These questions were summed up to the Social Trust Scale (0–30) and dichotomized into low (0–16) versus moderate/high social trust (17–30).

### Statistical Analysis

We estimated linear probability models (LPMs) with individual fixed effects to examine the impact of a change in WFH status (never versus partially/mainly/exclusively) on 13 binary health outcomes. All models were adjusted for survey waves. A *p*-value of ≤0.05 was regarded as statistically significant in all analyses. We present our results as marginal effects. All statistical analyses were conducted using Stata 18.0.

## Results

**FIGURE 1:**
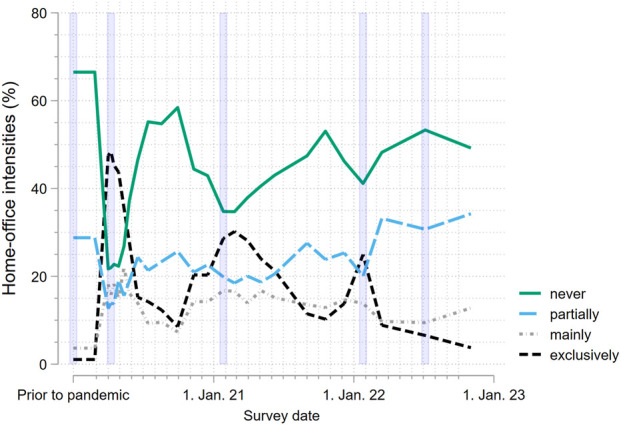
Working from home intensities over time (*N* = 651; COVID-19 Social Monitor, Switzerland, 2020–2022). Note violet bars correspond to the following five key points during the COVID-19 pandemic: (1) Before the pandemic: prior to the onset of the pandemic in early 2020 (February 2020); (2) Lockdown: during the spring lockdown in 2020 (April 2020); (3) Mandate I: following the implementation of the first working from home mandate (January 2021); (4) Mandate II: following the implementation of the second working from home mandate (January 2022); (5) End of measures: After the lifting of almost all COVID-19 protective measures ordered by the Federal Council in the spring of 2022 (Survey: July 2022).

The descriptive analysis (Figure 1) illustrates that, even well after the lifting of the mandatory COVID-19 WFH measures, the proportion of tertiary-educated employees working from home across all WFH intensities remained high relative to pre-pandemic levels. Specifically, the percentages prior to the pandemic versus November 2022 were as follows: 1% versus 4% working exclusively from home, 3% versus 12% working mainly from home and 27% versus 33% working partially from home. This increase was accompanied by a corresponding decrease in the proportion of those who never worked from home (68% versus 51%).

The marginal effects of transitioning to WFH on all health indicators considered (Tables 2–4) are consistently small in magnitude and not statistically significant. Subsample analyses by age and gender were conducted. The results across subgroups were consistent with the main findings (available upon request).

**TABLE 2:** Marginal effects of transitioning to working from home (partially or more) on general and mental health: Based on linear probability models (LPMs) with individual fixed effects (COVID-19 Social Monitor, Switzerland, 2020–2022).

​	Poor quality of life	Poor subjective health	Frequent stress	Heightened strain
At least partially working from home	0.000	−0.002	0.001	−0.001
​	[−0.008, 0.007]	[−0.009, 0.005]	[−0.023, 0.025]	[−0.017, 0.015]
*N*	651	651	651	651

95% confidence intervals in square brackets. All models adjusted for survey wave.

**TABLE 3:** Marginal effects of transitioning to working from home (partially or more) on physical health: Based on linear probability models (LPMs) with individual fixed effects (COVID-19 Social Monitor, Switzerland, 2020–2022).

​	Headache	Neck pain	Back pain	Sleep problems	Lack of energy
At least partially working from home	0.016	0.002	−0.002	−0.006	−0.001
​	[−0.012, 0.044]	[−0.026, 0.031]	[−0.029, 0.026]	[−0.034, 0.022]	[−0.032, 0.031]
*N*	651	651	651	651	651

95% confidence intervals in square brackets. All models adjusted for survey wave.

**TABLE 4:** Marginal effects of transitioning to working from home (partially or more) on health behaviour and social trust. Based on linear probability (LPM) models with individual-fixed-effects (COVID-19 Social Monitor, Switzerland, 2020–2022).

​	Complete physical inactivity	Frequent online-gambling	Frequent use of sleeping pills/sedatives	Low social trust
At least partially working from home	−0.017	−0.005	−0.000	−0.011
​	[−0.037, 0.004]	[−0.012, 0.002]	[−0.007, 0.007]	[−0.049, 0.028]
*N*	651	651	651	619

95% confidence intervals in square brackets. All models adjusted for survey wave.

## Discussion

Our study analysed how WFH affects the health of employees with a tertiary degree–the demographic group with the highest likelihood of WFH feasibility and adoption. The descriptive results across all WFH intensities confirm the recent upward trend in WFH among tertiary-educated employees, thereby underscoring the importance of health-impact research in this context [3, 13]. Furthermore, we find no evidence of an effect of transitioning to WFH on any of the 13 health outcomes considered, including poor quality of life, poor subjective health, frequent stress, heightened strain, headache, neck pain, back pain, sleep problems, lack of energy, physical inactivity, frequent online-gambling, frequent use of sleeping pills and sedatives, and social trust.

To the best of our knowledge, this is the first large-scale quantitative study utilizing a high-frequency panel dataset to explore the effect of a shift to WFH on health in this demographic group in Switzerland. Accordingly, our study provides a benchmark for future research by encompassing a comprehensive range of health outcomes and employing an extensive panel structure that spans periods during which WFH became an option rather than a requirement. Moreover, our study contributes nuanced insights to the inconclusive existing evidence in this area, [1, 13–15, 19, 31, 32]. Our findings align with those of Heiniger and Höglinger, who reported a slight increase in physical activity and no significant effect of transitioning to WFH on neck pain or wellbeing among the general Swiss working population [1] as well as with the results of Staller and et al. The latter study found no differences in sleep efficiency, sleep duration, variability of sleep timing or social jetlag between individuals working from home and onsite in a German sample of students and employees [16]. Finally, in terms of social relationships, it is worth noting that a meta-analysis by Gajendran and Harrison found no generally detrimental effects of telecommuting on the quality of workplace relationships [10].

### Limitations

Nevertheless, the data at hand has its limitations. Firstly, while general, mental and physical health could be thoroughly analysed, health behaviour and social trust could not be examined as comprehensively. Specifically, three of the four indicators in the latter two categories were not collected in all waves of the panel study–leading to slightly lower sample size for social trust (*N* = 619). Therefore, caution is warranted regarding these results, even though they align with those for the other indicators. Future research should thus focus on expanding the evidence on the impact of a shift to WFH on various areas of health behaviour and social wellbeing.

Secondly, although our study examines a comprehensive range of outcomes, future research should explore additional aspects. To this end, the recent conceptual model based on empirical evidence developed by Beckel et al. provides a useful reference point, despite its broader focus on telework. It highlights three important dimensions that could not be integrated into our analysis: diet, substance use and work-family balance. The authors point to empirical evidence indicating that teleworking employees are at a significantly lower risk of poor nutrition and tobacco use. Similarly, a survey study conducted among US healthcare workers during the COVID-19 pandemic found that those working from home eat more but consume healthier foods [33]. A Japanese longitudinal study conducted in 2020 concludes that overall diet quality improved during the pandemic; however, it also highlights the negative association between childcare burden and healthy eating [34]. In contrast, a study focusing on the lockdown period (February to April 2020) in Northern Italy concludes that COVID-19-quarantien might worsen the quality of diet [35]. Regarding alcohol abuse, Beckel et al. report varying results depending on when telework was carried out. Results are also equivocal regarding whether telework is beneficial or detrimental to balancing work and family [5]. Therefore, incorporating nutrition, substance use as well as work-family balance into future research could yield additional valuable findings. Furthermore, in light of our findings, future research using an even more extensive panel would help improve precision and assess whether small effects emerge over longer time horizons. More extensive panel data would also enable disaggregated analyses of the health effects of different levels of WFH intensity, thereby providing more in-depth insights.

### Conclusion

Our analysis provides no evidence of an effect of transitioning to WFH on any of the 13 health outcomes across the domains of general health, mental health, physical health, health behaviour, and social trust. The absence of adverse health effects of WFH is an important finding for employers and policymakers aiming to provide flexible work arrangements without compromising employee health and health-related productivity losses.
