# Supplementary material for: Shaping Workspaces, Shaping Lives: Health Implications of Working From Home for Employees With Tertiary Education in Switzerland
Source: Int J Public Health. 2026 Feb 13;71:1608002. doi: 10.3389/ijph.2026.1608002 (PMC12945841; doi:10.3389/ijph.2026.1608002)
Supplement: Supplementary file 1 [file Table1.docx]

Table S1: Survey questions and variable operationalization (COVID-19 Social Monitor, Switzerland, 2020-2022)

| **Area** | **Variable** | **Survey question** | **Original response options** | **Dichotomization** | **Source** |
| --- | --- | --- | --- | --- | --- |
| General health | Poor quality of life | How would you assess your overall quality of life at the moment? | Very bad  Bad  Neither good nor bad  Good  Very good | 1: Very bad, Bad  0: Neither good nor bad, Good, Very good | Swiss Health Survey, 2017 |
| General health | Poor subjective health | How is your health in general? | Very poor  Poor  Average  Good  Excellent | 1: Very poor, Poor  0: Average, Good, Excellent | Swiss Health Survey, 2017 |
| Mental health | Frequent stress | How often have you experienced stress in the last 7 days? | Very often  Frequently  Sometimes  Rarely  Never | 1: Very often, Frequently  0: Sometimes, Rarely, Never | Swiss Household Panel, 2018 |
| Mental health | Heightened strain | How have you felt in the last 7 days?  Very nervous  So down or blue that nothing could cheer you up.  Calm, balanced and serene.  Discouraged and depressed  Happy | Always  Most of the time  Sometimes  Rarely  Never | According to Mental Health Inventory (MHI-5)  (0 – 100; BFS 2019)  1: High (0 – 52 points)  0: Middle/Low (53 – 100) | Swiss Health Survey 2017, adapted reference period to 7 days |
| Physical health | Headache | Did you have any of the following symptoms in the last 7 days? Headaches, pressure in the head or facial pain? | Strongly  A little bit  Not at all | 1: Strongly, A little bit  0: Not at all | Swiss Health Survey, 2017 |
| Physical health | Neck pain | Pain in the shoulders, neck and/or arms | Strongly  A little bit  Not at all | 1: Strongly, A little bit  0: Not at all | Swiss Health Survey, 2017 |

Table S1: Survey questions and operationalization (COVID-19 Social Monitor, Switzerland, 2020-2022) (continued)

| **Area** | **Variable** | **Survey question** | **Original response options** | **Dichotomization** | **Source** |
| --- | --- | --- | --- | --- | --- |
| Physical health | Back pain | Back or lower back pain | Strongly  A little bit  Not at all | 1: Strongly, A little bit  0: Not at all | Swiss Health Survey, 2017 |
| Physical health | Sleep problems | Difficulty falling asleep or staying asleep. | Strongly  A little bit  Not at all | 1: Strongly, A little bit  0: Not at all | Swiss Health Survey, 2017 |
| Physical health | Lack of energy | General weakness, tiredness, lack of energy | Strongly  A little bit  Not at all | 1: Strongly, A little bit  0: Not at all | Swiss Health Survey, 2017 |
| Health behavior | Complete physical inactivity | In the last 7 days: On how many days were you physically active for a total of 30 minutes or more, causing you to breathe somewhat harder?  Examples of such activities include sports, exercise, training, as well as brisk walking or cycling, either for leisure or to get from one place to another. Please do not consider physical activities at home or as part of your work. | Number of days (1–7) | 1: Physical inactive (0 days)  0: Physical active (1 – 7 days) | Wanner et al. 2014 |
| Health behavior | Frequent online-gambling | Please indicate how often you engaged in the following activities during your leisure time in the past 14 days. I have played gambling games on the internet. (e.g., online casino, poker tournaments, lottery, etc.) | Multiple times a day  Once a day  Several times a week  Less often than once a week  Never | 1: Multiple times a day,  Once a day,  Several times a week  0: Less often than once a week,  Never | New item designed for survey |

Table S1: Survey questions and operationalization (COVID-19 Social Monitor, Switzerland, 2020-2022) (continued)

| **Area** | **Variable** | **Survey question** | **Original response options** | **Dichotomization** | **Source** |
| --- | --- | --- | --- | --- | --- |
| Health behavior | Frequent use of sleeping pills/sedatives | How often have you taken sleep aids or tranquilizers (e.g., Imovane, Sonata, Valium, Ativan, Xanax, Temesta) in the past 14 days? | Daily  Several times a week  Once a week  Less often  Never | 1: Daily, Several times a week  0: Once a week, Less often, Never | New item designed for survey |
| Social trust | Low social trust | Would you say that most people can be trusted, or that you can’t be too careful in dealing with people?  Do you think that most people would try to take advantage of you if they got the chance, or would they try to be fair?  Would you say that most of the time people try to be helpful or that they are mostly looking out for themselves? | You can’t be too careful (0) – Most people can be trusted (10)  Most people try to take advantage of me (0) – Most people would try to be fair (10)  People mostly look out for themselves (0) – Most people try to be helpful (10) | According to Social Trust Scale (0 – 30, Breyer 2015)  1: Low (0 – 16 points)  0: Moderate/High (17-30 points) | European Social Survey 9 - 2018 |
